# Rush Medical College

**Published:** 1868-02-15

**Authors:** 


### RUSH MEDICAL COLLEGE.

### ANNUAL COMMENCEMENT EXERCISES — ONE HUNDRED AND TWENTY-SIX DEGREES CONFERRED.

### *ADDRESS BY THE PRESIDENT AND RESPONSE BY THE CLASS.*

### THE CLASS OF '68 THE LARGEST THAT EVER LEFT A WESTERN INSTITUTION.

### VALEDICTORY ADDRESS BY PROF. R. L. REA, M.D.

THE twenty-fifth annual commencement exercises of Rush Medical College were held Wednesday evening, February 5th, in the spacious lecture room of the college, located on the corner of Indiana and North Dearborn streets. The room was full to overflowing with students of this institution — now the largest in the West — and their friends, including a number of ladies, and the exercises were of a most interesting nature, and gave evidence of the high position which the college has attained.

### CONFERRING OF DEGREES.

The exercises were opened by prayer by Rev. Robert Collyer, D.D., and the degree of Doctor of Medicine was then conferred upon each member of the graduating class, one hundred and sixteen in number, by the President of the Faculty, Prof. J. V. Z. Blaney, M.D.

The following are the names of

THE GRADUATING CLASS.

Francis G. Arter,
James B. Armstrong,
James H. Barnwell,
A. W. Bosworth,
Hugh Brownlee,
James Barr,
James R. Barnett,
James H. Baker,
Amos Babcock,
Robert N. Barger,
William H. Christie,
Pascal L. Craig,
John Cassidy,
Henry A. Chase,
James M. Cook,
J. A. Carter,
F. Wallace Coffin,
John B. Draper,
Nelson A. Drake,
David L. Davidson,
Thomas A. Elder,
George W. Elkins,
John T. Foster,
John G. Frank,
Benjamin H. Freeland,
David M. Finley,
Frank Fifield,
William Flinn,
William J. Fern,
John A. M. Gibbs,
Lyman T. Goodner,
John H. Goodell,
Joseph B. Griswold,
Henry C. Gemmell,
Samuel R. Hicks,
Abrogene Holland,
Cyrus Heywood,
Fernand Henrotin,
Merritt Hurst,
William H. H. Hagey,
Byron Holmes,
Christian B. Hirsch,
J. Robert Haggard,
Walter L. Johnson,
Thomas C. Kimball,
Thomas N. Livesay,
Gershom J. R. Little,
Edmund L. Lathrop,
William A. Looney,
Louis B. La Count,
John G. McKinney,
Abraham Miller,
Ben. C. Miller,
Charles Muth,
Leonidas B. Martin,
James McClure,
John B. Moore,
Americus V. Moore,
Samuel P. McCrea,
Thomas C. McCaughey,
William J. Maynard,
Thomas C. Murphey,
Francis McGuire,
Charles A. McCollom,
Albert B. McKune,
Edward L. Mayo, Jr.,
James Moffitt,
Albertis P. McCullock,
William R. McMahan,
Garrett Newkirk,
John R. O'Reiley,
Charles T. Parks,
William Quivey,
William S. Pitts,
Joel Prescott,
John H. Peters,
Bennett A. Payne,
James Pankhurst,
William R. Page,
Joseph B. Rood,
J. Rodney Rundlett,
Wilhelm Reinholdt,
Antinous A. Rowley,
William S. Robertson,
Justin Ross,
E. H. Pardee,
John G. Riddler,
Corydon Richmond,

Royal Reed,
Harrison Steele,
Ebert A. Sharon,
Daniel Spittler,
Josiah T. Scovell,
John W. Shipton,
De Witt Clinton Smith,
S. E. Scanland,
John P. Seawright,
Oscar F. Seeley,
John F. Shrouts,
Dana B. Seger,
Charles B. Thrall,
D. H. Arthur Thrane,
George O. Taylor,
John E. Tuttle,
L. E. Towne,
W. Alphonso Wood,
D. Lindley Woods,
Mather S. Wheeler,
Thomas Audley Wakeley,
Charles A. Wheaton,
Richard M. Wigginton,
Hiram G. Wyckoff,
Rush Winslow,
James I. Wakefield,
Henry Joseph Warmuth,
Thomas J. Yount.

The *ad eundem* degree of Doctor of Medicine was conferred on the following gentlemen: Daniel C. Babcock, M.D., John W. Cowden, M.D., W. F. Hani, M.D., William Little, M.D., William N. Bailey, M.D., Abner Hard, M.D. And the Honorary Degree of M.D. on Joseph Van Dyke, of Illinois; Orpheus Everts, M.D., of Indiana; John Ten Broeck, M.D., of Illinois; Joseph Janvier Woodward, M.D., Surgeon U. S. A.; J. S. Bobbs, M.D., Indiana.

President BLANEY then addressed the graduating class, as follows:

GENTLEMEN,—The diploma which each of you has just received, gives to you the endorsement of this Institution that you are prepared to enter the ranks of a useful, an honorable and a humane profession, as peers of its older members. It confers upon you the honors of Rush Medical College with whatever of prestige it has earned by twenty-four years of earnest effort, as an exponent of the principles, the practice, and the ethics of legitimate medicine. That you have chosen this, from the many schools of medicine whose portals were open to you, as that from which you have preferred to receive your degree, is evidence of your recognition that its diploma is of value, as a certificate of proficiency, and is respected by the communities among which you expect to labor for professional position and emolument.

This diploma carries with it the authority of the State of Illinois, that you shall be legally recognized as legitimate practitioners of medicine. It assures you that the Faculty of the

College and the Board of Trustees are willing to entrust to you the reputation of the Institution, by placing you upon the list of its Alumni.

These favors and honors you have earned by complying with the rules of the College, and by passing satisfactorily the ordeal of its examinations; but the acceptance of this diploma entails, on your part, duties and obligations, which as men of honor you can neither ignore nor avoid. These obligations are: 1st, to the College; 2nd, to the medical profession; 3rd, to community; and, 4th, to yourselves.

To the College that, as an Alumnus, you will never detract from her reputation by swerving from the direct paths of legitimate medicine, and giving countenance, either directly or indirectly, to any form of empiricism. To the profession, that you will strictly observe and practice the rules of ethics established as landmarks by the general consent and approval of its leading minds. To community, that you will, as members not only of a learned but of a benevolent profession, respond cheerfully to the demands of suffering humanity, even to the sacrifice of your own comfort and pecuniary interests, and contribute your knowledge and aid freely to the conservation and preservation of the public health, the diffusion and enforcement of proper hygienic laws and regulations. To yourselves, that you will comport yourselves as honorable and upright citizens, upholding the right and fighting the wrong, subservient to the just legal authority of the recognized government under which you may reside, and respecting its authority; and more than this, that you will continue to be unremitting students, with an earnest endeavor to advance the liberal science to which you are now admitted as professed licentiates.

To these obligations, gentlemen, are you bound by no oath, but by that which is equally strong and sacred—the respect due to your own chosen Alma Mater, and to your own and her honor.

Had I, gentlemen, the gift of eloquence, I would endeavor to impress upon you the high aims and the ennobling aspirations in which the true votary of our noble science is free to indulge; but I should couple them with narration of the toil, the sacrifices, the heavy burden of responsibility which clings around and impedes the progress of the aspirant for fame and position in the ranks of the honored few among medical men. I might deter those among you who are timid from pursuing the course of life which they have laid out for themselves, but I would arouse the earnest and the brave to greater effort to accomplish noble deeds of enterprise and discovery in the yet untrodden paths of physiology, of pathology, and of the branches devoted to the practical alleviation of disease. I could point you to a destiny and promise for you a niche in the temple of fame, which the kings and magnates of all nations might envy.

Your preceptors have only opened to you the portal of the temple whose *adita* you are now commissioned to explore. Upon all sides and before you are mysteries to be unraveled and immense treasures of knowledge to be gleaned, but not by sluggards, not by the timid, but by the toilers, the self-reliant, and the brave. Among which of these classes will each of you rank yourself? Permit me to give you a password, which is a key to success, to fame, and to the respect of your fellow-men, and of posterity. That password is WORK. Keep it before you, carry it on your person, think of it, dream of it, but above all *practice* it. It is the secret "sesame" which, with a fervent reliance on the aid of a Providence which helps all those who will help themselves, opens the door to professional success, and exhibits the treasures which have been stored and retained for use of the fortunate explorer.

Remember that your success reflects honor, your failure disgrace upon this Institution and the Faculty who have to the best of their ability labored to induct you into the rudiments of your profession. By great effort of your Faculty this commodious edifice and abundant appliances for your instruction have been provided for you. The present course of lectures has been conducted under great disadvantages, from the unfinished state of the building at the beginning of the session, by which much serious inconvenience and additional labor was required of the Faculty so as to fulfil their own wishes, and in making the course complete in every respect. They are willing and are satisfied that the proficiency of the graduating class should be the test of the measure of success they have accomplished, and I am fully authorized by my colleagues in expressing to you, gentlemen, the great satisfaction which they have experienced from the constant and unremitting attention upon the lectures by the entire class, the harmony which has prevailed universally between the pupils and their instructors, the uniform gentlemanly deportment and respect for order, both as regards your conduct in the Institution and outside among the citizens, which have been marked features of the present session, and by the general high average of proficiency exhibited in the examinations of the graduating class of 1868.

We do not feel like parting with you, gentlemen, without this expression of our honest sentiments that you have done your part in sustaining them in their efforts, and of our further satisfaction that not a single death or case of serious illness has occurred among the members of this the largest class which has ever attended our College, and throughout this the longest session we have ever held. This we accept as good evidence of the health of the city, the sufficient ventilation and heating of our building, and the good moral character and proper observance of hygienic laws by the class at large.

We desire further to say to every graduate, that his diploma constitutes him a member of the Alumni Association of the Rush Medical College, which was organized at the opening of the session just closed, and that his frequent attendance upon the annual meetings which are hereafter to be held on each Commencement day of the College, is earnestly invoked, and will be accepted by the Faculty as the strongest evidence of continued attachment to the Institution with whose interests and welfare they have this evening become forever identified. And not only then, but at all times, all the advantages for study which the Institution now furnishes or may hereafter provide, are open to each one, and he will always have a cordial welcome to the halls of his Alma Mater.

I can not close without a word of remark to the under graduates. You are all, gentlemen, familiar with the disadvantages under which the Faculty have labored from the impossibility of providing for the arrangement of their respective Cabinets and the Museum in the earlier part of the course. These disadvantages will not obtain at the opening of the next session, and I am authorized to state that many and important additions to the means of illustration will be made in each department, and a complete arrangement of all the cabinets will be effected before the opening of the next session, and if it is felt by you that the course of instruction for the present year has given satisfaction, we may safely anticipate that increased facilities will warrant the officers of the College in promising a still more satisfactory course of instruction to the class of 1868 and '69.

Walker L. Johnson, of the graduating class, responded as follows:

Sir,—In behalf of my classmates I acknowledge the honor conferred upon us by the Trustees of this College, by you and your associates.

We fully recognize your right to demand of us, on the receipt of these diplomas, that we shall at all times strictly adhere to the practice of legitimate medicine, refrain from all things which savor of empiricism and charlatanry, and shall ever recognize and be governed by a strict code of medical ethics.

This, from the oath of Hippocrates, shall be a guide for each and all of us: "My life shall be pure and holy; into whatever house I enter I will go for the good of the patient; I will abstain from inflicting any voluntary injury, and from leading away any, whether man or woman, bond or free."

In their name I will say, that the time will never come when this college will be ashamed of this class.

We shall always be proud of our Alma Mater; her interests are our interests; we shall ever labor to sustain her in her present proud position, and will always be prompt to defend her from both open and insidious attacks. This is not the last time this college shall see us. You will hear from us frequently. This is our home; this the head center of medical truth. We are the radii of the circle, over which the waves of scientific truth travel from hence throughout time and space. And, sir, I speak as if each one of my fellows in arms were speaking of himself when I say, with this diploma in my good right hand, and with this indomitable heart, there shall be a record of that truth, both in teaching and practicing, that shall redound to the permanent fame of this great institution. And may my tongue cleave to the roof of my mouth, and this right hand forget her cunning, if I forget Rush Medical College!

Doctors are teachers. We now are both students and teachers, every one of us. Both our pupils and ourselves shall make pilgrimages to this as our medical Mecca. These are sacred walls to us; this is our temple. But there is being built a temple, a prouder temple, to scientific truth, and this not of bricks and mortar. This institution is part of its foundation; aye, I might almost say its chief corner-stone. Now and henceforth we are, and will be fellow-workers, co-laborers in this great work with you, sir, and your colleagues. We are proud of the age in which we graduate: the most glorious period in the history of medicine. This age, when the fair temple of medical science, from which the debris of the fallen battlements of ignorance has been swept away, stands on the firm foundation of anatomy and physiology.

In behalf of my fellow students I tender the faculty our warmest thanks. Our relations, as instructors and pupils, have been those of unalloyed pleasure, and although eager to enter the arena to battle for public favors, and to assert the truths we have been taught, the pleasure we anticipate is mingled with regret at leaving you and this place. But we leave feeling that we are better men from contact with such instructors; that we have imbibed not only medical knowledge, but also much from those high attributes of the human mind, humanity and philanthropy, which are no less essential to the success and existence of the physician and gentleman.

### PROF. REA'S VALEDICTORY.

One of the commonest mistakes of mankind is concerning people. We seldom grade a man rightly. There are so many and such varied points to be considered in reckoning men, that but few hit their exact value. In deciding the merits of a piece of mechanism, an acre of land, or a horse, there is little difficulty; but in man we have qualities and circumstances to estimate entirely absent in Dexter or an avenue lot.

We either call a man weak when he is strong, or strong when he is weak, or very able when he is medium, or feeble when he is tolerable. Two classes we invariably misjudge — our friends and our enemies. The one is colored by the rainbow hues of attachment; the other clouded by the sombre shades of indifference or hatred. This erroneous adjustment of human worth depends on one of two causes — either a lack of ability on our part to properly estimate our subject's surroundings, how much he has to contend with mentally, morally or physically, in life's grand make-up, or an unwillingness amounting to preconception, bigotry or narrow-headedness to do so if we could.

We put a man up or down in the scale of merit, more often from the latter than the former cause, influenced by a moral color-blindness which eliminates all the shades not needed to form our ideal spectrum. Could there be invented a biological dynamometer, it would create much surprise and some fluttering, as the gaudy birds of paradise and the slimy polywogs of human society changed places.

In the present brief interview I desire to consider two or three of the erroneous views held by some concerning the profession, which embraces so large, respectable, useful, and talented a body of men as that to which you have been so recently formally introduced, hoping that our attention being directed to these misapprehensions we may be the better able to correct them.

No man, or set of men, can expect to be properly understood by every one, and I do not intend touching my complaining strings very strongly, nor to say that we are erroneously judged by every one or in every thing.

Conceding, as we must, that we have our defects in common with every other class of men, yet it seems to me there is a little additional zest given to the ridicule heaped on a physician, the sarcasm a little more bitter and pointed, the arrow prepared to pierce his foibles, follies, or faults, dipped a little deeper in the pungent, biting vocabulary of calumny and derision, and the appearance he presents, when the public tongue gets through with him, a little more deplorable than any other class to which the aforesaid public devotes its exclusive attention for any considerable length of time.

Perhaps this results from the expectations being too great, excited by the *assumed* pretensions of our branch of the liberal professions, assumed, not by us, but accredited to us as a ground for detraction when our success falls short of their absurd anticipations.

It need hardly be premised that we hold a place among the handiworkmen of human society second to none. The interests confided to us are as dear as the dearest, the trusts reposed the most gigantic that fall to the lot of man to guard.

We stand as man's vanguard in the unequal contest with death. We also fight in a contest where we are certain at last to be defeated. However well or adroitly we conduct our forces, the fiat has gone forth that death must conquer, however long we may turn aside his shafts.

Often, from the weakly efforts of the enemy, or from skillful management of the defence in our hands, parrying the well-aimed thrusts as we stand side by side with our patient, and, perhaps, receiving the blow aimed at him, we succeed in baffling him so long that the confidence of those trusting attributes omnipotence to us; and when the stately tree that has so long breasted the storm, comes crashing to the earth, it shakes not only the ground on which it falls, but sends a reverberating shiver through the forest, creating distrust in those whose power, though long successful, has succumbed to the inevitable, and the distrust is usually proportioned to the past confidence.

Then, dissatisfied, slow to feel that we are less than the ideal of their own creating, a despondent apprehension gains the place of former faith, the throne they made and the attributes by which they surrounded you, are swept away like gossamer by the breath of death.

One of the most invidious and disparaging mistakes concerning our profession, and one that stands at the beginning of many others, is the opinion held by some, that it is a matter of the most sovereign indifference what medical man or system they employ, and send the servant for a doctor as one would for a stick of firewood, and decide on a system as he would the color of his wife's dress or the direction he would drive.

This is most easiest shown in the readiness they change physicians, taking up with any conjurer, pathy, man or woman, who possesses and gives the assurance they desire, however unreasonable their pretensions may be, or stupid *they* may appear in embracing such a transparent delusion. Their want seems to be to employ some one professing to heal, expecting at the magic pass or word to take up their beds and walk, whether it come from learning or ignorance. In one of the reasons for this we find another erroneous idea entertained by the unskilled, that disease is a unit, like a lion roaming through the streets, and remedies the guns to destroy it; that medicine, in other words, is an exact science, like mechanics or mathematics—a given amount of disease in one end of the scales can be counteracted by a given amount of medicine in the other. This is a most unfortunate error, and observations in medical practice would lead us to infer that it is not confined to those outside the profession. Medical science is made up of certain deductions from a given number of facts, and the practice of medicine experiences with those deductions. We must first examine and understand the mechanism we desire to repair or preserve. After ascertaining the nature, qualities, and uses of the different parts, we can tell when each is acting naturally and usefully, and, knowing when it is doing so, tell when it is excessive, deficient, or deranged in its action, and the cause of it. We would find by experiment, and that alone, what supports would stay any given part, what material might be added to or taken from any other part; what kind, quality, and shape of instrument would be suited to cut away an offending part or adapt it to others.

Medicine, then, is a science and an art, one consisting of a knowledge of the organism, and the materials used in its regulation, the other consisting of the ability to apply these agents, or remedial measures, to the cure of disease.

A knowledge of man, and the agents that influence him, shows that he is made up of organs co-related, that forces exerted on one part react on another, and no organ so remote that it may not be disturbed. How, then, can disease be an entity? A blow on one part may cruelly afflict another, and this widening influence derange or destroy the whole body. Load one wheel of your wagon too heavily, it gives way, the axle bends or breaks, twisting the bed, loosening the firm-fitting joints, and, if not speedily corrected, surely and quickly destroys the usefulness of this necessary adjunct of civilization. So disease induced in the stomach by an overload, speedily allows the brain to sway down, loosening the smooth monuments of joint, muscle, and mind, and were there no more chances for, or kinds of relief, than are offered by its inanimate prototype, the mysterious midnight trips to unfrequented localities, for which we get unjust credit, would be rendered superfluous, for they (the objects) would be plentier than missing men's bones in our janitor's cauldron; our roads would be strewn with old rack and ruins, ulnar and radial spokes, scapular hubs, vertebral ridge poles, rib bows, cranial and pelvic feed-troughs, while here and there a bush would boast a cutaneous cover to shelter the belated carrion crow, whose arduous labors and press of business prevent its nightly return to the bosom of its family.

This idea of unity, carried to its legitimate inference requires a given medicine for each disease, and the idea stands at the basis of at least one system of medicine. Medicine from this is given with the idea that it goes through all the channels of the system in search of the invader, to overtake, strangle and destroy it.

The disastrous effects of such a belief, and its consequent results in practice, had better be left to the undertaker, whose legitimate business it is to deal with them.

And yet we find men indifferent, not to say reckless, in not investigating the relative value of men or systems, and the vast probability is, their opinions are guided in deciding the most vital questions in regard to life's best essence, good health, and all that makes life desirable, by those that in ordinary business they would not deign to notice.

What apology could a reasonable man offer for such carelessness in selecting his medical attendant? He would suggest want of time, want of suitable knowledge to decide, and perhaps come to my first assertion, if he had no other to offer, that it made little difference, so he was a doctor.

Would he select a clerk for his employ with equal carelessness? Would he employ a lawyer to conduct his most trivial case without evidence of his ability? If he did not understand law enough to decide on the merits of his attorney, would he not devise some way to find, before he trusted him with finances, whether he were suitable to be trusted ? He would know whether he had success in practice, had a diploma, and was conceded by the bar to possess talent, industry and honesty.

But how is it often when the question comes up, Who shall be my physician ? Seldom selected until he is absolutely wanted, no time can be taken to decide on merit, and one is thrust on him, the quickest to be had, a sense of petty obligation binds him, and perhaps to the veriest pretender. Too often the decision is arrived at by the suggestion of some neighborhood gossip, to whose electioneering efforts her favorite owes what of success he has.

Suggestions, again, of friends, often influenced by any thing but sterling worth, serve to fix his easy decision, and the whims and caprices of some old splinter of feminine antiquity balances against all his better judgment. How readily he forgets, if he ever knew, that a majority of people, however intelligent or rational they may be on general subjects, are the blindest and most unreliable on subjects connected with health. Medicine is viewed and treated by them as a great mystery. Entrusted once to priests, it now seems removed but one step from the mystery of divinity, and this superstitious reverence of its patients requires tame submission to its mandates, the more mysterious the more impressible and valuable. Few persons escape entirely this influence. All remember the feelings of childhood as we looked at the old family physician, and believed him little beneath the Saviour in his power to control disease, whose welcome presence was perfect security, and whose face was watched as an index of fate. And now carrying this feeling into life in a mitigated form, it stands as a great barrier to the use of sound judgment and common sense, so essential in any thing else.

Instead, then, of endorsing professional similitude and its miscellaneous value, it should be a matter of the most conscientious and pains-taking investigation who shall be guide and guard through the labyrinths of disease. He must also remember that men of like ability and professional qualifications yet differ in many desirable points, and while there may be no objection, even on account of his attainments or system of practice, there might be differences in secondary qualities which were invaluable to him. Of direct medical qualification he can of course judge but little, but observations will soon disclose to him — if he exercises half the tact and effort he does in his business — who does or does not understand what he professes. He will soon be able to decipher the cabalistic signs nature has marked in his features, and decide what she has placed behind to support his pretensions, whether he belongs to the grand army of helpers or the detested gang of hurters.

Another misapprehension concerning our profession, and one that tends to weaken the influence and lighten the esteem felt for the worthy medical man, is the expressed belief of a very large part of the community that the avocation we follow tends to blunt the finer feelings, sear the sensibilities, and render us indifferent to the feelings of others — in other words, sinks our sympathies out of sight in our science. It is the old story over again, told of an undertaker engaged in coffining a man, who was interrupted by his unexpected return to life, and, wanting to know the meaning of present preparations, "Don't bother me," said he, continuing his work, "I'm busy."

Next to the fault of an unqualified physician, would I set the one of heartlessness. I presume the fully matured opinion of three out of every four persons you meet, is that physicians attend and care for their patients, with the same feeling a carpenter constructs his box, or an apothecary compounds his medicines.

This idea is so old and well formed, that I fear no words of mine can interfere with the cataract of opinion against us. If you take a pig and mix a portion of madder daily with his swill, at the end of a certain time his bones, if examined, will be found tinged with the madder. Instead of their pearly white color, you will find the reddish color of the pigment, and no washing will displace it, until the part is replaced by new bones. So with the majority of the world. They have fed on the idea of the merciless nature of physicians, till it has become deposited in their bones, and forms a part of their deep, unchanging nature. The worst element in this opinion, when well believed, is not the influence it compromises in community, but it reacts on the holder to his serious disadvantage.

Sympathy is an element of cure, it is a remedy for disease, and only those possessing or appreciating it can tell how potent. All know how different the feelings, as appreciated by different persons, under trials of any kind. One takes you by his kind, loving, tender, and hearty sympathy, lightens at once your burden, by such apt, happy, cheerful, and hopeful words, that he lifts half the sorrow, and paints the other deep in the glowing colors of hope and cheerfulness. The other comes bustling with sharp, short, crisp, mechanical words, the expressions of sympathy freezing on his very lips, frigid as adamant, repulsive as death, plunging the shaft deeper instead of extracting one pang from your sorrow. Take these two men as physicians in the sick room, one inspires confidence and courage; the other disheartens, creating opposition and distrust.

What I maintain is, that this kind, humane sympathy, this innate devotion to the interests of others, this all-essential of the true physician, is possessed by our members in a degree, and to an extent held by no body of men of equal numbers on the broad face of God's earth.

If not, why do we as we do? Can it be for money?

Could men be hired for money to undertake and undergo what we cheerfully do? Could you obtain for a few paltry dollars the services of a person to expose himself to storms on nights when life and limb are risked in the venture? Is there a man to be found who, influenced by purely selfish considerations, would freely expose himself to the horrors of small-pox, yellow fever, and cholera, in their most virulent forms? Are there men among us who would hire out to go to Sandusky, Norfolk, or New Orleans, to supply the help decimated by death, where the grim monster seemed to have set his seal on all living? No, there is something deeper and better in the hearts of the brave men who have done this, and we claim that the higher and nobler motives which actuate them stand forth in living characters and practical forms in the glorious band of men, whose lives are devoted to alleviating the ills man credits to death. Money is well. It is a great comfort; but there must be some thing stronger of the good than money to call men out into such broad exposure. Man's selfishness forbids and must be counteracted by benevolence.

The mistaken idea of indifference arises partly from the necessary behaviour of surgeons. The surgeon who promptly and scientifically performs his operative duties soon secures names ranging from cool indifference to brutality. No matter how thoroughly competent and careful, yet he is simply Saw-bones.

Why, say they, he cuts as though he was amputating a stick of wood! Why shouldn't he? It is merely mechanical — his patient off to dreamland — shall he substitute crying for cutting, and let the well-springs of sorrow for the misfortune which has deprived a patient of a member gurgle up, trembling the hand he stretches to save? Standing as the arbiter to decide between worlds for him, should he stand simpering, while he should be staying the tide that ebbs his life away? Familiarity with suffering does not necessarily callous the sensibilities, and this is particularly true where our associations with it occur, as in ordinary medical practice, among our friends and neighbors.

In the medical department of our art occurs the greatest amount of censure, and more indignation is excited against us, as a class, by complainers with whom there is little the matter, who, in proportion to the smallness of their ailings, are importunate to be heard, and woe to the doctor who has too much to do to hear the most minute and unimportant details of family history, ramified back generations, with all the delicate elaboration of an oft-told tale — the more restless you become, the more animated they, at last centering by one grand culmination in an "impression of the heart," or a sense of "empty goneness," *your* only regret being that it is not entire. It is never supposed that a physician is too busy to listen to such details, and, if there should be such dereliction, the complaints of the victim will do more to establish his reputation, by their continued, persistent repetition of the offence, than a dozen real causes for the opinion.

This opinion of our calling I consider very unfortunate, for it not only tends to detract from one of our most meritorious qualities, but injures the confidence which stands as the centre pillar of our fabric. What can we do without the confidence of our patrons? and what would undermine that so quick as to doubt your kindness, however much they might admire your professional ability?

Another mistaken idea concerning us is that we supply material for dissecting purposes by exhuming it. Nothing can be more erroneous. We are happy to acknowledge the aid of our city papers in dispelling that illusion by the timely suggestion that the large numbers of missing men of whom you have seen and read, have found their way into our dissecting room. It seems strange how it could have been discovered, as we have never told any one, and it is an axiom or proverb, I don't know which in this case, that "dead men tell no tales." The fact is we have been overrun with missing men during the present session. A man, from some financial or domestic reason or fancy of his own, desires to be missing. He applies for admission to our missing department. We advise, expostulate, remonstrate, but if it all fail we take him in, but like the spider and the fly, he never comes out again. This will account for the crowd seen during the winter on our front door step. These were all missing men, and a remarkable fact in this connection is that when they came inside they could not be seen.

It is well for the city and surrounding country that they concluded to be missing, or some one else might. The way we manage our supply for the missing department, when volunteers fail, is this: We see a person that is well adapted to our purposes, and we send a polite invitation to join us, and he never fails to comply; we have a *pressing* way of urging him. We indulge our most trivial fancies in such things. If an operatic prima-donna appears, and our curiosity awakens as to how she manufactures notes of such fascinating quality, we send for her, and she is missing. This will explain the great number of artists who are missing from America within a year or two. Then, a ballet girl must have some peculiar anatomics about the tiny feet she spirals on so witchingly. This will account for the sudden disappearance of an entire troupe lately from our city, as we had to indulge the curiosity of our large class on the cause of their sprightliness. It is said, in further proof, that little pattering feet, like rain on a roof, can be heard gliding, fairy-like, through these halls in the small hours, and the irradiate form of "outdone" steps tiptoe from back to back of these seats, singing, with beckoning hand, "Come." Any one desiring to verify this can do so at three o'clock any very dark morning, by coming stealthily up the front steps alone. There are many persons in our city yet in danger. Intending, as we do, to investigate every anatomical peculiarity of men and women, not a few, we are thinking of inviting a dozen or two clergymen, in order to examine their eyes, to discover by what peculiarity of make the visual organ of one, looking at the identical page, sees things so entirely different from all the rest. One lawyer's tongue must be examined, to discover by what peculiarity in its construction he can tell the truth; and a similar organ in a lady I have heard of, who don't talk back; and also of another I *know*, who has some barbed arrangement about hers, so that when she pierces a person with it, she can drag out the largest reputation; and of an uncertain number of physicians' hands, who never write an unsuccessful prescription. Our General will be apt to receive attention, to discover what valvular arrangement he has about his mouth which allows him to draw such constant volumes into it, while the most ingenious devices of the most cunning politician can draw nothing out of it. I may add, as an explanation of the lack of the heaps of bones that would necessarily accumulate, that we sell them to the Western Elastic Sponge Company, who convert them by an undiscovered process into their commodity, and many are no doubt now enjoying the ballet troupe in another luxurious form.

One other misconception I shall allude to—the counterpart of the first—the tendency to distrust all remedies, or medical infidelity. There are fashions in medicine as in mantua-making, and our mantua-makers have their busy seasons as well as the other kind, often finding it as difficult to becomingly dress their customers.

Different forms, sizes, and complexions require different colors, styles, and combinations. How absurd, as sometimes happens, a selection is made because becoming another, and when applied to them the short dress or pumpkin-seed bonnet is simply hideous. In the medical fashions we have similar ludicrous blunders. For some the simplest and silliest fashions are most suitable — they admiring, select substantial, valuable habiliments, exposing themselves to the ridicule of the *elite* of fashion, while another, whose honest form has never indulged the fancies of fashion, is betrayed into decorating herself with the blazing flummery she has admired on the head of some brainless beauty, till she looks like the elaborately decorated queen of an insane asylum. You can no more prevent a silly, senseless, lackadaisical, affected, aping, brainless woman or female man from patronizing a diluted form of nonsense in medicine, than you can interpret the intentions of our municipal phantom. In medicine, as in religion and politics, mankind seek their level. It is needless to expect to find all agreeing in any one way or method.

We notice certain classes of mind tend to certain beliefs. It is said in one form of religion that a majority of the membership patronize a certain medical fallacy. I have noticed, also, that some of the more evangelical forms of religion tend to the more substantial and true in medical belief. Many men are so organized that they tend to all kinds of infidelity, while others tend to credulity for a similar cause. Ignorance, like muscular fibre, is of two kinds, voluntary and involuntary, with perhaps the third kind — mixed. The voluntary embraces neglected opportunity, and want of ability to obtain necessary knowledge, to accomplish something we have undertaken. The other kind, involuntary, covers that we need but can not know. This latter comes home forcibly to us, ignorant as we are of the causes of things which, if we knew, would be the key to many other unexplained facts.

Take the example of the nervous diseases which afflict the body, yet leave no traces after death, as we can discover, that they ever existed. The most violent form of neuralgia, that rendered the life a burden it would not take, leaves not a mark to show that ever a twinge of pain passed along its innocent-looking threads. In insanity what do our most intimate researches for the cause of this most fearful discord in Nature's thousand-stringed harp give us for our trouble? How certainly we know the brain to be the seat of mental action, and yet the connection of mind and matter is such that the exponent of the mind, the medium through which it acts, its work-shop, may totter and tumble, and yet leave not the slightest trace of disease's strongest touches. The deep secret of mind's relation to matter evades the most searching stroke of the keenest scalpel.

Take the blood, the great carrier of good and evil, freighted heavily with concentrated destruction, where shall we find the chemist whose cunning filter is equal to its separation? or who can analyze the morbid element, tell us how it acts, and give us the antidote to counteract and destroy it?

One strong point made against us, is involuntary ignorance, and we acknowledge the fact, but by no means engross it.

The profession of medicine has always been regarded as a suitable target to discharge the sharpest calumnies against, when ignorance was the theme. That it suffers from this cause none deny who are acquainted with its composition. All occupations and professions do likewise, and we suffer no more nor less than they. Why, then, do we get credit for this vast amount of latent information? Why are we distinguished above the other liberal professions in this invidious particular? and why this distrust founded on it? One reason occurs to me in the difference, honest and dishonest, among its members.

Persons unacquainted with the principles of our science, can not see how we can differ upon any subject which, in their opinion, should be so straightforward. Disease, in their eyes, is a unit with its definite remedy, and when one physician cures a disease with a given agent, any other one differing must be wrong; and if both cure, then the whole thing must be a phantom. The differences of doctors has become proverbial, and I shall not contend that they have always been justifiable or profitable.

But a freedom of thought, the exercise of our strongest powers of reason, and most careful observation and analogy are alone consistent with the most successful and rightful practice of the art of healing, no matter who it causes us to differ with. We must be allowed the same diversity of means to accomplish an end as is granted to the other professions.

If a child is affected with croup, and one physician uses an emetic of ipecac; another one of sulphate of zinc, alum, lobelia, or tartar emetic; while another uses a bandage of cold or hot water, it must not be concluded that there is a mistake about the disease, and that neither understand it, because they adopt different means to circumvent it. It would not be said so of any other person employed, if in his discretion he showed a disposition to evade the beaten path, in hopes of finding some more easy or efficient way to do you good service; it would be taken as an evidence of genius and estimated accordingly; but in the physician it is presumptuous, it is tampering with health, and can not be tolerated. A lawyer requested to foreclose a mortgage may choose one of three ways to do it — by sale under the power of the mortgage, by a bill in chancery, or by *scire facias;* or to collect an amount due you, he may bring an action of debt or assumpsit; if your property is illegally taken or held he can bring an action in trespass, trover or replevin, the different remedies applied being varied by the circumstances of the case.

Religion or the hygienics of the soul may be resolved into this simple statement for its object: Man's goodness and purity, and the glory of God. Notice the diversity of means used for accomplishing these coveted conditions. How much space would be required to express in their simplest form the points of difference between the religious sects, and their methods in detail, for correcting man's moral deformity. One applies water to him; another him to the water; another does neither; another believes in an instantaneous change of heart; another a gradual; another in neither. One believes in the Old Testament, another in the New, and another in neither, and yet if we take the test given by the highest authority for pure and undefiled religion, we find the most beautiful and pure lives coincident with all these teachings. As in our profession, so in this, we can not always distinguish causes from coincidences, and yet the fact remains. In this advocacy of liberty of opinion and freedom of election of means for given ends, I do not wish to be understood as proving too much, for I no more include the bastard offshoots of our profession than our legal friends include among themselves shysters, the fungus excrescences that deform, by their pestilential presence; the ancient and honorable calling they *mis*-represent, or would our clerical brethren Mormonism incantations and free-loveism, the low, degrading and diabolical inventions of bad men, as substitutes for the pure and simple truths enunciated in the best of books. These illustrations of diversity of method could be multiplied largely, but enough has been given to show that we have counterparts, and that it stands as no excuse for want of faith in medicine; and the commotions about the difference of doctors, whether in law, medicine, divinity or philosophy, only indicate an intelligent discrimination on the part of the men you select as your counsellors and guides — and not distraction, distrust and demoralization. I would not be understood, however, as endorsing all the differences that arise between the members of our profession.

One of the most suicidal things our members are guilty of is the bitter, unprofitable, wordy wrangling in which they often indulge, sometimes with seeming plausibility on both sides, but conducted in a spirit any thing but amiable, candid, or fraternal. The effect is, that people conclude that when persons differ so widely, persistently, and fiercely, that truth lies in neither. One of the greatest causes of these differences is faulty observation. The observer don't use his eyes correctly. He is either blind, or cross-eyed, sees nothing, or sees one thing where another should be. By a primitive mistake in determining the disease, he builds a pyramid of additional mistakes in remedies for the supposed disease, and perhaps caps the climax by condemning the authorized treatment for the affection. Take a common symptom, such as pain through the chest. Now, you know this is but a single symptom, but may be an overwhelming one. It may be from one of several diseases. It may come from disease of the liver, lungs, pleura, spine, or rheumatism. Suppose a careless or ignorant man undertakes to name and treat the affection, and substitutes one for the other, confounds a pain through the chest from an overloaded stomach with a similar pain from pneumonia, and uses the active, depressing treatment of the latter for the former disease, and persists, as such usually do, to cover his mistakes. What will his opinion likely be of medicines? or what will be their curative effects? Suppose he mistakes causes instead of diseases. Take dropsy as an example. We have it, as you know, coming as the result of several derangements—as the kidneys, liver, blood, or heart—and substitute one of these for another as the cause of the affection, and treat accordingly, the disastrous results need not be suggested to you.

Then, there arises an endless dispute over the effect of remedies, set down by one as invariably successful in a given class of diseases, and by another, if not hurtful at least inert. Take the war between prominent members of our profession on calomel and alcohol, what must the uninitiated think to see one member declaim against these articles as useless—worse than useless, and poisonous—while another challenges him to produce a case they have killed by them, and produce their scores that have been saved by their administration? Here is serious discrepancy and want of care in observation in some body, when prolonged and united experience is thus unceremoniously upset.

Newman Hall, the celebrated English divine, who recently visited America, having occasion to remark the infrequency of drunkenness among us, said he had seen but three or four intoxicated men during his entire sojourn. Bishop Clark came to a similar conclusion concerning Europe, having seen, as he said, during five months' experience there, but five men and one woman drunk; congratulated Europe and bewailed America as giving more inebriates than any similar number of people on the globe.

Here are men talented, and capable in their profession, yet how slow we are to believe that there were but the limited numbers in either hemisphere intoxicated, and within easy reach of the unaided vision of the distinguished authors of these extravagant but erroneous compliments. We should circumscribe the area of ignorance, and particularly the kind I term voluntary; the other occupies far too wide a field to allow any additions from neglect, and with all the care we can exercise we are imperfect enough; and disease, often when pursued to its most remote fastnesses, evades our most searching efforts to discover its nature or treatment.

Mr. Stanley, one of the most eminent scholars and writers on the subject of bones, once amputated the limb of a young lady, for what proved afterwards to have been a hysterical affection of the knee-joint—this after making the most careful and thorough investigation. These discrepancies between physicians gives a rare chance for its opponents to deny its efficacy on account of its alleged uncertainty. Why, say they, doctors themselves can not agree concerning the effects of their remedies; where are their claims to our confidence, when they even admit that they can not always be certain what effects will follow their administration? We do not pretend infallibility, for our science is not an exact one; and the crumbs of comfort they seize from this seeming defect can be equally applied to every other not exact—and yet there are many aids to increase the prejudices arising from this cause.

A medicine may be exact so far as the influence is concerned, and yet some unseen influence may turn aside its good effects, and render unavailing our bravest efforts. These occult forces, uncounted among the elements of disease, often beyond the reach of the most careful and educated observation, are parts of the grand reef upon which the small boats of our fleet founder. Men with such weak tethering may "Autocrat a breakfast table," but can't captain the tiniest craft that skims our sea, in the great crusade against life's grand enemy. Difficulties of this kind surround and frustrate us when success seems easiest, and yet uncertainties are not novelties in any pursuit of life. How few callings are exempt from them.

The farmer, as he spreads broadcast his grain and labor, forms little idea what harvest will bring. The herdsman little knows what disease may ruin his brightest hopes at the most unexpected moment. The merchant, as he despatches his precious cargo, never takes the return invoice till the sails of his giant servant are safely reefed in the home harbor. The minister, as he pours forth his burning words fresh from the deepest recesses of his feeling heart, never can reduce the result to mathematical calculation, and is as ignorant as ourselves, no matter how deep the dose of sulphur, what psoral counteracting influences are at work to neutralize his most earnest, true and hearty labors. Even the law, the profession founded in precedent, is proverbially one of the most uncertain of all. You canot predict with certainty the issue in the simplest case, whether, when you bring suit for your fee of three hundred dollars, employing the best legal doctor, that the defendant may not perjure himself, transparently to judge and jury, and yet they may reduce your claim one-third. We are not exempt from uncertainties in our expectations of the effects of our remedies, but it is not peculiar to us. We have no means of analyzing a remedy that will tell beforehand its effect on the human system. This can only be determined by experiment, and, when its general and ordinary action is ascertained, there are sometimes those hidden stops that interfere and counteract what we have a right to expect. This is seen occasionally, in the form of an idosyncrasy, as some persons can not come in reach of the dust of ipecacuana without an attack of asthma, as others suffer from nettle rash from eating strawberries; and yet, shall we cease to smother the scarlet pellets in the saccharine-lacteal congelation, because one person in a thousand feels constricted about his throat, and millions of pins titilate his skin; or shall we cease to employ ipecac because, occasionally, instead of producing emesis it produces asthma; or sleep's step-brother, chloroform, because in an infinitesimally small number of cases the patient exchanges celestial dreams for heavenly realities? I think not. What a glorious harvest this uncertainty that mistily surrounds our practice gives to the guerillas, camp-followers, and buzzards who watch the sick as they stagger from physician to physician, with some helpless form of disease, expecting their prey as hope dies out and courage at last fails. Well they know when a man becomes distracted by the thought of hope lost he rattles the medical dice box with little care what turns up so it *promises* relief. All that a man hath will he give for his life, and with what desperate clutches he grasps at the least straw that appears able to delay his exit from his coveted home.

How sorrowful to see the utterly frantic efforts, and the vapid illusions used to bolster up the sinking hopes of some hopeless invalids. Perhaps a man of strong intellect still unstrung by the grimy embraces of the fell destroyer, now a willing subject in the hands of some repulsive, ignorant, designing old crone, fed by her confident promises, while the hectic flush that mantles his cheeks is the rosy ornament that marks him for the eternal blooming. All that a man hath will he give for his life, even to his manhood, self-respect, and better judgment. What a crop of prejudice, bigotry, and superstition is fostered by this uncertainty, leading to mistaking a coincidence of cure for its cause.

It is one of the unbearable embarrassments you will be called on to endure. The gold chain around the neck will take the place of your best treatment for quinsy; the red woollen string around the little finger will cure more nose-bleed than all your *materia medica.* The blood taken by a splinter from an aching tooth, and driven into the north side of a persimmon tree, will cure more toothache than all your creosote. Warts can be cured by a stolen bacon rubbed over them and buried under the eaves, better than any remedy at your disposal, except you take a woollen string and tie as many knots in it as there are offenders, and bury it in the same place. Some remedy would long ere this have been discovered for hydrophobia had not the magic touches of the mad-stone rendered such efforts useless.

The inflamed palate, popularly supposed to have fallen, can better be restored to health and place by drawing on eleven hairs on the crown of the head, than any thing you can devise, except you advise them to do as a patient of mine insisted she had—swallow it.

A gentleman of this city gave me as a fact, that an impression prevailed in the region in which he was raised, that when one of a family died of a given disease, if another were attacked the disease might be arrested by exhuming the body of the deceased relative, and causing the invalid to inhale the fumes formed from burning a part of the body of the deceased similar to that affected in the invalid, which many years ago was actually tried in the family of a relative of his, but, singular as it may appear, the consumption with which she was affected moved on uninterrupted.

But sad and discouraging as are these mistaken views of us, I do not think a true philosophy requires we should sit down and bewail our lot, in being misunderstood and misrepresented. A more excellent way occurs to me in endeavoring to so represent the profession in our conduct, qualifications, and successes, that a better respect will be accorded to us. The false impressions named are not always without some show of reason. When we so far lower by our voluntary ignorance and indifference the standard of our professional qualifications, and defeat instead of defend the efforts of nature to re-establish herself on the throne of health, thereby leveling ourselves with the counterfeits, what wonder we should be mistaken for them! Is it to be wondered at, that when we convert ourselves into the hard, pitiless, inexorable creatures some do, instead of softening by contact with suffering, that we are set down as cruel and unfeeling? or that the rankling enmities brought about by our internecine contentions should stagger the faith of those who should trust us? or that an unskillful application of remedies, with the irregular result, should create sus ceptibilities to bad impressions of legitimate medicine, and tendencies to depart from the good old way and follow the gods of the heathen? But while there are reasons for these many opinions of us, there are also remedies. We have, as a faculty, been industriously and faithfully trying to place them in your hands during your term of pupilage. The only true remedy is the proper education of the members who are to mould and represent this great branch of the liberal profession. We have tried, as faithful guardians of the public, whose most serious and vital interests hang on your strength as medical men, to thoroughly qualify you to skillfully perform the arduous duties you are soon to assume, and at the same time to bar the entrance against all over whom a doubt could arise. We most willingly and heartily acknowledge the enthusiastic co-operation you have accorded us in this grateful task. We are led by our own experience to hope great things of you.

Instead of coming wheezing, breathless, and exhausted by your efforts, you came bounding into the arena, fresh and anxious for the contest, eager for a tilt with death, to try the temper of the Damascus with which you have been furnished. Bright as it gleams, symmetrically as it curves, handsome as it hangs, it must be judiciously handled to be made valuable.

You will have to guard yourselves in all ways to make the most of the talent confided to you. Temptations inumerable lurk by the wayside to destroy, and the physician is exposed as no other can be; it is inseparable from his duties and relations, and will require all the moral courage of which you are possessed to defend yourself against those trials incident to your position. Often times the influence of others over you will be your weak point, and when a generous nature renders you the most companionable and welcome, you must be on guard against pernicious influences, and keep your hearts closed against friends and foes, when to allow them to enter paralyzes your self-control.

One of the first pieces of gratuitous advice given you by anxious and interested friends is, to get married. I advise you to do no such thing, unless you are so situated that you can properly care for the partner you select. It is one of the first thoughts in the hearts of most young men, and the burden of advice from all the old ladies, who will assume to advise you in matters of success; and this will be particularly true of those who have six or eight pairs of black eyes, of the feminine gender, about twenty-seven years old, and the more so if their chins begin to recede from their collar-bones, and their sterno mastoid muscle begins to ridge the surface.

Don't listen to them. There is no more necessity for, or obligation resting on, a physician to get married, than a carpenter, lawyer, or a dentist, what ever people may say about it.

Marriage is all well and natural, but entangled as most of the beginners among you will be, in the meshes of financial strategy, remember, two birds in a net only disturb and batter each other with their wings, without benefit to either in their efforts to escape. First establish yourself, and then catch your bird if you can. Arms may answer for a while, but they must be used to drive the wolf away from the door, not to perpetually fondle the lamb. Palpitating hearts in your situation are pleasant—when you get a chance to prescribe for them. I am offering this to all the unmarried, unmoneyed members, fully aware how few will take it.

Out of all I see before me, I doubt not that for a large share of those single there is a little muffled drum beating to night, a delicate little hand, with beautiful taper fingers, pressing in anticipation the one in which she laid hers, with her little flutterer now beating triumphant marches through his future life. She will stand, like the sweet little angel she is, waiting the signal far down the prairie as the locomotive rounds the curve in sight, blessing the cars and track, engineer and conductor, brakesman and newsboy—all because they have brought you safe home again. The quiver of delight as she sees you first with those sparkling eyes, half ashamed of the total wreck they have made of her efforts at concealment, kindles blushes to add new evidence, if you needed any, of the devotion of this splendid little piece of dimity you intend to follow my advice and marry—as soon as you are able. But don't forget, if she loves you till she can't see, and you love her till your sight is somewhat dim, wait till the certain day dawn of success endorses the wisdom of your enterprise.

But if you are bound to marry her any way, give her my love and the congratulations of the faculty, and tell her you have graduated in the largest and best class that ever left Rush Medical College, and you guess you will take the chances.

Don't forsake your chosen calling. There is nothing in the education you have received here that especially qualifies you to measure a yard of tape or an acre of ground, and the sacrifices bear no proportion to such uses. The disposition to leave the profession, I am pleased to say, is rare among our alumni, and of the few who do it, it is better they should. If you have selected your profession with the care you should have exercised at first, I advise you, as you value success, to stand firmly to your chosen avocation, relying on scholarly attainments and correct deportment to secure you the patronage you desire, however dark the future seems to spread before you. Change distracts and destroys a man's courage.

Bulwer has it:

> "The man who seeks one thing in life, and only one,
> May hope to win it ere life be done;
> But he who seeks all things where'er he goes
> Only reaps from the hopes which around him he sows,
> A harvest of barren regrets."

Were I to sum up in one sentence, advice upon which to found your hopes of future success, it would be to feel and manifest a genuine, true, hearty, manly interest in your patients. I do not mean that stepmotherly nervous anxiety, looking akin to retain instead of restore, but that devotion to the interests of your patients that needs no interpretation but your face. Possessing this you will possess all things, the confidence and love of your patients, second only to the blissful realities of an entered heaven.

Gentlemen Graduates: This evening closes the pleasant relation of teacher and taught that have so pleasantly bound us together. Henceforth our paths diverge, and many of us part to meet no more this side the river.

Parting from friends always leaves a pang, the severity of which is proportioned to the pleasure of past associations. Taking this as a guide, the feeling that arises as we separate will be that of sincere, unmingled sorrow, for, as far as I am aware, not one act of yours has tended even to moderate the happiness we have always felt as your teachers, or the confidence we rightly feel in you. Your duties have been performed cheerfully, promptly, and successfully, and standing as you do, the largest class of graduates, if I am not mistaken, that ever left a Western institution, the spirit of emulation that has characterized you has far exceeded any of your predecessors—and I am happy to testify to the result in the superior qualifications that make you, I believe, the best as well as the largest.

We can congratulate ourselves, also, in the fact that death has not only not entered our beautiful temple' but that no serious case of sickness of any kind has occurred during this session, and I am spared the sad allusion I was compelled to make when last I had the honor of appearing in my present position.

It is related of the great musical composer, Beethoven, that "after having finished his great and only opera, his grand conception of character could find no counterpart in the real world. He could find no one to personate his Leonora. The musical public and his friends urged the old man impatiently to give the result of his magical genius to the world. He obstinately refused, and finally silenced them by threatening to burn it if it was ever mentioned to him again. A young actress of wondrous power, with deep blue eyes, passionately and fathomlessly deep, possessing magnetic power, had just returned to Vienna, Wilhemina Shroeder, who had been performing in neighboring cities, and though scarcely seventeen years old, the most extravagant prophecies were made of her success. During his morning walk he invariably met this charming girl, who, unknown to him, was unnoticed. One day, during a thunder storm, she managed to meet him, and, going up to him, muttered some unintelligible words. Surprised to see her there, and at such a time, he bent down and asked her what she was doing out in such weather; had she lost her way? A sweet voice answered faintly but quietly, "I only wished to see you." "See me! For what can you want to see me?" "Your Leonora." He started; asked her name; she told him how she had tried to attract his attention; had to-day ventured to speak to him. "Did uou not see the storm? Were you not afraid?" Afraid of but one thing," said she; "that you would not give me your Leonora." Such artless and unaffected simplicity and gentle determination did their work. "Come to me to-morrow morning," said he, "I think I have found my Leonora." She went, received her instructions, gathered from his masterly hand all the accessories to a proper rendition. The part in which the test of her power was, where Floristan, a prisoner of the Inquisition, is confined in a subterranean dungeon, whose location has been unknown for two years to Leonora, his wife, who at last, by strategy, gains admittance to him, but does not reveal herself. A plan is made to murder Floristan, by one of the officers of the prison, to conceal his oppressions; Leonora—or Fidelio, as Beethoven afterwards named the character—was by when the plot was about to be executed. At the critical moment she shielded her husband with her own body, and uttering this cry, "Kill first his wife," saved him.

A few weeks after this, when the opera was brought out, Beethoven sat in a little box near the stage. As the opera progressed, the sweet, powerful tones of the young singer reached his almost closed ear (for you will recollect he was almost entirely deaf), and cheered him. As the climax approached, he arose in almost feverish excitement; he gasped for breath, his form quivered, and his eyes were fastened on the singer's lips. For a moment she seemed to hesitate; suddenly she drew herself up in a truly magnificent manner, and dashed the vibratory thrill with the greatest passion into the much moved auditors. A miracle seemed to take place. The powerful effort penetrated every barrier, and reached the master's ear. Suddenly the whole work mirrored itself in the overwhelming sound, as the universe mirrors itself in a raindrop. The feeling of happiness overcame him, and this man, so strong in affliction, so accustomed to sorrow, fainted."

You, gentlemen, are each a Floristan, about to be imprisoned in the intricacies of our art, who have wooed and won your Fidelio (medicine), and are to devote yourself to her as your future wedded wife. If you have been judicious and honest in your selection, and will truly and faithfully cultivate the virtues she possesses, she will prove to you a self-sacrificing, invaluable shield against all the malignant shafts sent against you, and at the right moment, will interpose herself to rescue you from the thickest dangers that gird your path through the mazy labyrinths of an imperfect science. We, as the authors of the work in which you are about to engage as characters, will stand as no indifferent spectators, while you perform your assigned role. The overture is ended, the call-boy at his place, the prompter waiting, the last bell has rung, and with what fevered anxiety do we see the curtain rise, and each of you move to his proper place. Only when you are fairly started will we breathe freely, and be quiet enough to enjoy. When your trial comes, and the faithful Fidelio interferes to save you, then, though we will be deaf from distance, let the loud sweet tones of your triumphs reach us even here, and fill us with the boundless, unspeakable rapture that comes from work well done, committed to high, deserving, and appreciative hands. In behalf of the Faculty who have taught, and the Trustees who have honored you, I bid you God speed, and good-bye.

At the close of the address the benediction was pronounced by Rev. Mr. Lathrop, and the exercises were then concluded.

